# Abundance of domestic mites in dwellings of children and adolescents with asthma in relation to environmental factors and allergy symptoms

**DOI:** 10.1038/s41598-021-97936-7

**Published:** 2021-09-16

**Authors:** Krzysztof Solarz, Anna Obuchowicz, Marek Asman, Wacław Nowak, Joanna Witecka, Jolanta Pietrzak, Marta Marek, Aldona Łonak, Izabela Stadnicka, Bernadeta Hajduga-Staśko

**Affiliations:** 1 Department of Parasitology, Faculty of Pharmaceutical Sciences in Sosnowiec, Medical University of Silesia in Katowice, Katowice, Poland; 2 Department of Pediatrics in Bytom, Faculty of Health Sciences in Katowice, Medical University of Silesia in Katowice, Katowice, Poland; 3 Department of Molecular Biology, Faculty of Pharmaceutical Sciences in Sosnowiec, Medical University of Silesia in Katowice, Katowice, Poland

**Keywords:** Epidemiology, Paediatric research, Asthma

## Abstract

Exposure to house dust allergens, mainly from domestic mites, is an important cause of allergic reactions in sensitized asthmatic patients. A total of 63 dust samples were collected from 16 flats in Bytom (south Poland); in each flat a person (age 4–17 years) suffering from bronchial asthma lived with his/her family. Mite density was calculated as the number of specimens per g of dust. The results were compared with household features and the data were statistically analyzed. In total 566 mite specimens were isolated, including 526 members of the family Pyroglyphidae (93%). The dominant species were *Dermatophagoides pteronyssinus* (60% of the total count) and *Dermatophagoides farinae* (32%). Pyroglyphids were found in all mite positive samples (68%) of which 35% also contained non-pyroglyphids, including glycyphagids, cheyletids and gamasids. The results suggest associations between the density of some mite taxa (per g of dust) and the following indoor environmental factors: presence of pets, number of inhabitants, coal-stoves as a type of heating, cleaning frequency, higher relative humidity, presence of flowers and PVC windows. The severity of asthma seems to be associated with the numbers of *D. farinae*, total domestic mites and live mites per g of dust.

## Introduction

The number of cases of atopic allergies has increased in industrialized countries. There is increasing evidence that house dust belongs to the inhalant allergens that most commonly provoke allergic diseases in humans. Exposure to house dust allergens is an important cause of allergic reactions in sensitized asthmatic patients. The main allergenic part of house dust is domestic mites, whose density depends on various indoor environmental factors, both biotic and abiotic^1–7^.

House dust mites from the family Pyroglyphidae (Acari: Acaridida) are recognized as the most important risk factor causing allergies in indoor environments^6,8–11^. Three species—*Dermatophagoides pteronyssinus*, *Dermatophagoides farinae* and *Euroglyphus maynei—*are reported to be the major allergen sources in house dust^9,12–16^. House-dust-mite allergens occurring in an indoor environment are the most important agents of respiratory diseases in humans, including asthma^17–19^.

The most common species of mites found in dwellings in Poland is *D. farinae*, which grows optimally at 70–75% relative humidity (RH) and 23–30 °C^4,5^. The critical humidity for the survival of mites ranges from 40 to 45% RH^1–3,12^. Therefore, the installation of systems reducing indoor humidity in dwellings has been suggested as a method of controlling the house-dust-mite numbers. It was also suggested that indoor environmental conditions are important for the development of allergic diseases with manifestations in the respiratory system, including atopic asthma^1–3,12,17^. For example, damp housing promotes infestation of house dust mites, which increases the risk of sensitization and the development of allergic diseases. Various measures to reduce house dust mite growth have been proposed, including enhanced cleaning and airing, and the use of mattress encasings^17^.

Sensitization to house dust mites is a major independent risk factor for asthma and other allergic diseases, such as perennial rhinitis, atopic dermatitis, urticaria and/or oculorhinitis^4,20^. However, the importance of exposure (types of housing) in progression from sensitization to developing asthma is far less clear. To date there are about 15–20 published studies from all over the world that have investigated the relationship between household characteristics and mite allergens levels. Some of the studies were too small to enable multivariate analysis and some only included patients with asthma^21^. Although data collected on household characteristics varied greatly between studies and despite the fact that building materials and techniques differ in various parts of the world, some common trends have emerged^11,16,21^. Knowledge of the household characteristics which is associated with high mite allergen levels is important in a public health context^16,22,23^. To date, a wide range of abiotic indoor environmental factors in dwellings has been investigated for their influence on pyroglyphid dust-mite populations, in the hope that limiting factors may be exploited in control^1–3,5,7,17,21,24^.

Several recent studies have shown that many patients with an allergy—who for the first time have been observed with asthma—were exposed to relevant indoor allergens^11,21,25^. Previous reports of microscopic examination of mites in dust from patients’ houses (flats, dwellings) demonstrated regional differences in recovery of mites and the species identified^9,10,12,16,26^.

The aim of this study was to investigate house dust mite occurrence in relation to types of dwellings and indoor environmental conditions, possibly affecting the development of asthma in children and adolescents with an allergy to house dust mites, living in Bytom, near Katowice, in south Poland.

## Materials and methods

The study was performed in 16 flats occupied by 16 persons suffering from bronchial asthma, with ages ranging from 4 to 17 years. All subjects were selected from the patients of Department of Pediatrics in Bytom (Medical University of Silesia in Katowice), which have agreed to participate in the study. All these patients were previously diagnosed with an allergy to dust mites. According to GINA 2006 classification, 1 person was diagnosed with severe asthma, 9 with moderate, and 6 with mild asthma. Moreover, 4 persons suffered from atopic eczema and 9 from perennial allergic rhinitis. Allergy to *Dermatophagoides* spp. was confirmed according to the current standards^27,28^. The results of skin prick tests with allergens Der p 1 and Der p 2 were positive, and the following levels of IgE were detected: against Der p 1—very high (> 17.5 IU/ml) in 12/16 patients, high (> 3.5–17.5 IU/ml) in 2/16 patients, moderately high (> 0.7–3.5 IU/ml) in 2/16 patients and against Der p 2—very high in 12/16 patients, in 12/16 patients—high, in 2/16—moderately high, and in 1/16—low (> 0.35–0.7 IU/ml).

The examined dwellings were located within the same urban or suburban area of the old and big city of Bytom in Upper Silesia, south-eastern Poland, and were investigated regarding the content of the house dust mites and different household environmental factors. A total of 63 dust samples were collected from the flats and examined for the presence of allergenic mites. The samples were collected from beds, upholstery, wooden furniture and floors, carpets, linoleum, as well as from other places, such as bedclothes and walls. A surface area of 1 m^2^ at each sampling site was hoovered for 2 min. Next, the samples were weighed and analysed for mites, as previously described by Solarz^5^.

All mites were mounted in Hoyer's medium on slides, and the species and life stages were determined with the aid of a compound microscope. Mite density was calculated as the number of specimens per 1 g of dust. Mites that were alive at the time of sampling could easily be distinguished as intact (undamaged) and because of their plump and/or white appearance. Next, results of these studies were compared with household data of the persons suffering from asthma. Information on various parameters which could influence the numbers of mites was obtained by questioning the residents. These parameters (explanatory variables *x*) were: age of the building, locality (urban or suburban), type of building (close/compact or freestanding), type of building construction (housing block, concrete or brick), level of dwelling (first floor or higher), area occupied by each flat, size of family, type of window frame (PVC or wood), type of heating (central, electric, gas, oil or coal-stove), type of floor (carpet, plastic or wood), level of humidity, presence or absence of domestic flowers/plants, presence or absence of pets, place and time (month) of sampling, and weight of samples. The following factors characterising the sleeping accommodation of the examined asthmatic patients were included in the analysis: type of sleeping accommodation (bed/mattress or couch), type of bedclothes (feather beds, quilts, blankets and pillows), and temperature in the bedroom. Additional parameters analysed were frequency of cleaning and housewife (employed or not). The criterion variables (*y*) on the other hand were: number of mites per gram of dust (total or live), in relation to particular pyroglyphid mite species, total house dust mites (Pyroglyphidae), total domestic mites (including non-pyroglyphid allergenic mites—acarids, glycyphagids), and total mites (including other taxa). The data obtained was analysed using the Kolmogorov–Smirnov test, the Spearman’s rank correlation test, Mann–Whitney U test, Wilcoxon signed-rank test and χ^2^ test.

## Results

### Overall results

The age of buildings varied from 10 to 105 years ($\overline{x}$ = 51.61). The weight of samples ranged from 0.01 to 2.4 g ($\overline{x}$ = 0.24). The results obtained are presented in Tables 1, 2 and 3. It should be stressed that 43 (68.25%) samples were positive for mites out of a total 63 examined dust samples (Table 1). A total of 566 mite specimens were isolated, including 526 members of Pyroglyphidae family (92.93%) (Table 1).

**Table 1:** Species list, dominance, frequency and abundance of mites found in house dust samples from dwellings of the examined asthmatic patients in Bytom (south Poland).

Mite taxa	Dominance		Frequency			Abundance^3^
	N	%	n	%^1^	%^2^	
*Dermatophagoides pteronyssinus*	339	59.89	21	33.33	48.84	5.38
*Dermatophagoides farinae*	183	32.33	29	46.03	67.44	2.90
*Euroglyphus maynei*	3	0.53	2	3.17	4.65	0.05
*Gymnoglyphus longior*	1	0.18	1	1.59	2.33	0.02
*Acarus siro*	1	0.18	1	1.59	2.33	0.02
*Tyrophagus putrescentiae*	1	0.18	1	1.59	2.33	0.02
*Lepidoglyphus destructor*	10	1.76	3	4.76	6.98	0.16
*Glycyphagus domesticus*	1	0.18	1	1.59	2.33	0.02
Cheyletidae	22	3.89	10	15.87	23.26	0.35
Mesostigmata	5	0.88	2	3.17	4.65	0.08
Total mites	566	100	43	68.25	100	8.98

N—number of specimens, n—number of positive samples, ^1^ in relation to the total of samples examined (n = 63), ^2^ in relation to samples positive for mites (n = 43), ^3^ mean number of mites per 1 sample in relation to all samples examined (n = 63).

**Table 2:** Combinations of the occurrence of particular pyroglyphid and non-pyroglyphid mite taxa and their relative frequency in the examined dwellings of asthmatic patients in Bytom (south Poland).

Combinations of mite taxa	Number of samples positive [Proportion]^1^
Samples containing only pyroglyphid mites	28 [0.44]
DP	7 [0.11]
DF	18 [0.29]
DP + DF	1
DP + EM	1
DF + DP + EM + GL	1
Samples with pyroglyphid and non-pyroglyphid mites	15 [0.24]
DF + AS	1
DF + TP	1
DF + ChE	1
DP + ChE	3
DF + Gam	1
DP + DF + GD	1
DP + DF + Ch E	3
DP + LD + ChE	2
DP + DF + Ch E + LD	1
DP + LD + ChE + MES	1

^1^in relation to a total of samples examined (n = 63) (all proportions can be calculated by the reader, only those > 0.1 are shown), DF: *Dermatophagoides farinae,* DP: *Dermatophagoides pteronyssinus,* EM: *Euroglyphus maynei,* GL: *Gymnoglyphus longior,* AS: *Acarus siro* complex, TP: *Tyrophagus putrescentiae,* GD: *Glycyphagus domesticus,* LD: *Lepidoglyphus destructor*, ChE: *Cheyletus eruditus,* MES: Mesostigmata.

**Table 3:** Relationships observed between housing conditions and the prevalence of mites in dust samples from dwellings of the examined patients in Bytom (south Poland). Results of the Spearman’s rank correlation test analysis (*p < 0.05). Part I.

Number of mites (per 1 g of dust)	Dwelling conditions examined: R Spearman						
	Type of building	Type of heating	Age of building	Pets	Family size	Flat size	House wife
*Dermatophagoides pteronyssinus*	0.40*	0.31*	0.42*	0.23	− 0.30*	− 0.29*	− 0.18
*Dermatophagoides farinae*	− 0.38*	− 0.13	− 0.38*	0.01	0.16	− 0.15	− 0.12
Pyroglyphidae (total)	− 0.04	0.10	0.01	0.07	− 0.75	− 0.37*	− 0.23
Domestic mites (total)	− 0.05	0.10	− 0.004	0.28*	− 0.10	− 0.36*	− 0.25
Total of mites	− 0.05	0.10	− 0.004	0.28*	− 0.10	− 0.36*	− 0.25
Live Pyroglyphidae (total)	0.14	− 0.04	0.21	0.18	− 0.23	− 0.06	0.01
Live domestic mites (total)	0.14	0.25	0.18	0.18	− 0.24	− 0.26*	− 0.21
Live mites (total)	0.14	0.25	0.18	0.18	− 0.24	− 0.26*	− 0.21
Live *D. pteronyssinus*	0.25	0.03	0.35*	0.26*	− 0.39*	− 0.13	− 0.13
Live *D. farinae*	− 0.11	− 0.09	− 0.13	− 0.26*	0.17	0.09	0.21

Type of building: block of houses [1], tenement-house [2], brick house (old) [3]; Type of heating: central [1], electric/gas/oil [2] or coal-stove [3]; Age of building: in years; Pets: no pets [0], fish/reptilians [1], birds [2], mammals (cats, dogs, hamsters, rabbits) [3]; Family size: number of inhabitants; Flat size: area occupied by each flat (surface in square meters); House wife: not employed [1], employed [2].

### Domestic mite fauna

The two dominant species were pyroglyphids, *D. pteronyssinus* (n = 339; 59.89% of all mites) and *D. farinae* (n = 183; 32.33%). In addition, 3 specimens of *E. maynei* (0.53%) and 1 female of *Gymnoglyphus longior* (0.18%) were collected (Table 1). Pyroglyphid mites were found in all mite positive samples, but 28 contained species exclusively from this family (44.4% of all samples examined and 65.12% of samples positive for mites) (Table 2). Also only 15 samples contained non-pyroglyphid mites (23.81% of all samples examined, 34.9% of samples positive for mites), including members of the families Glycyphagidae (*Lepidoglyphus destructor*, *Glycyphagus domesticus*), Acaridae (*Tyrophagus putrescentiae*, *Acarus siro* complex), Cheyletidae and of the order Mesostigmata (Table 2). Among 25 samples with a single mite species, 18 samples contained *D. farinae* and only 7 of them *D. pteronyssinus.* 9 samples were co-inhabited by two species of mites, 7 types of the species composition were noted, and most frequently the combination of *D. pteronyssinus* and a member of the family Cheyletidae (3 samples) occurred. All of these combinations of mite species composition, which were observed in dust samples from dwellings, are listed in Table 2. This comparison also includes mites of other taxa. No mites were found in 20 samples (31.7%). The majority of domestic mite species occurred with low frequency (Table 1). Therefore, for these species and for some dwellings, the median values of numbers of mites per gram of dust were 0.0. *D. farinae* was the most frequent species and it was found in 29 samples (46.03% of the total count and 67.44% of samples positive for mites) (Table 1), whereas *D. pteronyssinus* was found in 21 samples (33.3% of the total count). The difference was statistically non-significant (Yates corrected χ^2^ = 3.01, p = 0.083).

### Abundance of mites

*D. pteronyssinus* was the most abundant species per 1 sample (Table 1), whereas *D. farinae* was more abundant per 1 g of dust. The mean numbers of mites per 1 g of dust were approximately 127.6, 69.9, 197.9, 209.2 and 209.3 for *D. farinae, D. pteronyssinus,* total pyroglyphids, total domestic mites and total mites, respectively. Whereas the mean numbers of mites per 1 sample in relation to all mite positive samples are presented in Table 1. The numbers were approximately 5.4 and 2.9 for *D. pteronyssinus* and *D. farinae*, respectively. Densities (numbers of mites per gram of dust) of the total mite population, total domestic mites, total pyroglyphids or particular pyroglyphid mite species varied from one dwelling to another, and from one location to another within the dwellings. In bedrooms, the allergenic mites, both house dust mites and total domestic mites, were most abundant on pillows and on quilts, whereas they were much less numerous on sheets. Generally, *D. farinae* was more abundant in bed dust samples than *D. pteronyssinus.*

### Relations between the household characteristics, bronchial asthma and domestic mites

The highest number of mite species was found in a 105 year old house, 3 species in 70-year old, 50-year old and 10-year old houses. The persons who lived in these houses showed the symptoms of moderate, episodic, severe and moderate asthma respectively. The mean age of the buildings where the asthmatic patients with moderate asthma lived was 59 years, and with episodic asthma 58 years. Most patients suffering from episodic asthma (5/6 persons; 83.0%) lived in detached houses on the city outskirts, built of concrete slabs or bricks. Most patients suffering from moderate asthma (6/9 persons, 67.0%) lived in high-density housing in the center of Bytom (tenement houses).

No *D. pteronyssinus* was found in the samples from 3/4 of the flats located in the buildings with concrete slabs as well as in the tenement houses. Only 3/16 of the flats were located above the first floor (II, III, IX). No mites were found in the dust samples collected from the first two flats, two mite species were recorded in the third flat, however the number of mites in 1 g of dust was fairly small (max. 12.9). Only two flats were heated by coal stoves; *D. pteronyssinus* and *D. farinae* were present in both flats and the number of mites in 1 g of dust was high (max. 933.4 and 380.0). The samples of dust taken from 4 flats with insulated windows with plastic frames showed various numbers of mites; in two flats they were high (max. 466.7 and 5000/1 g), and in two low (max. 24.1 and 80.0/1 g). Additionally, the type of floors and signs of dampness in the flats did not show any connections with the species and concentration of mites in the dust.

The majority of the data actually obtained was not distributed normally (Kolmogorov–Smirnov test; p < 0.01 or p < 0.05).

The number of pyroglyphid mites per gram of dust (both total and live mites) in the examined dwellings was significantly associated (Wilcoxon signed-rank test) with the number of inhabitants (family size) (p < 0.001), coal-stoves as type of heating (p < 0.001), higher cleaning frequency (p = 0.000004), dampness (p < 0.001), presence of flowers (p < 0.001), employed housewife (p < 0.001) and PVC windows (p < 0.001), whereas bedroom temperature was significantly associated only with the number of live pyroglyphids per gram of dust (Wilcoxon signed-rank test, p < 0.001). Number of *D. farinae* per 1 g of dust was significantly associated (Wilcoxon signed-rank test) with presence of pets (p < 0.001), coal-stoves as type of heating (p ≤ 0.01), dampness (p = 0.04), presence of flowers (p ≤ 0.005), employed housewife (p ≤ 0.02) and PVC windows (p ≤ 0.01).

Higher relative humidity in flats significantly influenced only the higher numbers of live *D. farinae* and live *D. pteronyssinus* per 1 g of dust (Mann–Whitney *U* test; p = 0.036 and 0.0074, respectively). Free standing houses had significantly higher number of *D. farinae* per gram of dust (Mann–Whitney *U* test; p = 0.0051). Typical beds (with mattresses) contained significantly higher numbers of *D. farinae*, live house dust mites and live domestic mites per 1 g of dust (Mann–Whitney *U* test; p < 0.01, p = 0.012; p < 0.05, respectively). Moreover densities of *D. farinae* (per gram of dust) were associated with suburban localisation of the house, with PVC windows and with flats located in the brick houses (Mann–Whitney *U* test; p < 0.05, p < 0.005 and p < 0.01, respectively).

The severity of asthma was significantly associated with the numbers of *D. farinae*, total house dust mites, total domestic mites and live house dust mites per gram of dust (Wilcoxon signed-rank test; p = 0.0051, p < 0.001, p < 0.001 and p = 0.00012, respectively). Rhinitis was significantly associated with the total house dust mites and total domestic mites per gram of dust (Wilcoxon signed-rank test; p < 0.001, in both cases).

The results of the Spearman’s rank test for correlation between some abiotic and biotic indoor environmental factors (housing conditions) and mite prevalence and density in the examined dwellings suggests associations between density of some mite taxa (per gram of dust) and the following abiotic or biotic indoor environmental factors: type and age of building (*D. pteronyssinus* with old brick houses, *D. farinae* with block houses and new houses), type of heating (*D. pteronyssinus* with coal-stoves), presence of pets (positive influence on densities of domestic or total mites and live *D. pteronyssinus*), family size and flat sizes (positive influence of smaller families or flats), suburban localisation (on *D. farinae*), place of sampling (beddings and beds on pyroglyphids, domestic and total mites), higher weight of sample (with exception of live *D. farinae*), type of sleeping accommodation (mattresses on live pyroglyphids and couches on *D. farinae*, total pyroglyphids, total domestic mites or total mites), absence of flowers/plants (on live *D. farinae*), lower level/floor of a flat (on *D. farinae,* total pyroglyphids, total domestic mites and total mites), type of windows (PVC on *D. farinae* and wooden on live pyroglyphids), higher cleaning frequency and higher moisture. Other correlations were nonsignificant (p > 0.05) (Tables 3, 4 and 5).

**Table 4:** Relationships observed between housing conditions and the prevalence of mites in dust samples from dwellings of the examined patients in Bytom (south Poland). Results of the Spearman’s rank correlation test analysis (*p < 0.05). Part II.

Number of mites (per 1 g of dust)	Dwelling conditions examined: R Spearman						
	Build-up	Localization	Place of sampling	Month of sampling	Weight of sample	Type of bed	Type of floor
*Dermatophagoides pteronyssinus*	− 0.24	− 0.03	0.002	0.15	0.04	0.02	− 0.08
*Dermatophagoides farinae*	0.39	0.28*	0.21	0.15	− 0.15	− 0.38*	− 0.12
Pyroglyphidae (total)	0.10	0.14	0.31*	0.26*	− 0.13	− 0.27*	− 0.22
Domestic mites (total)	0.01	0.15	0.31*	0.28*	− 0.13	− 0.27*	− 0.22
Total mites	0.01	0.15	0.31*	0.28*	− 0.13	− 0.27*	− 0.22
Live Pyroglyphidae (total)	0.05	− 0.13	− 0.10	− 0.22	0.50*	0.28*	− 0.19
Live domestic mites (total)	− 0.02	− 0.11	− 0.03	− 0.03	0.29*	0.13	− 0.24
Live mites (total)	− 0.02	− 0.11	− 0.03	− 0.03	0.29*	0.13	− 0.24
Live *D. pteronyssinus*	− 0.10	− 0.06	0.05	− 0.05	0.33*	0.22	− 0.18
Live *D. farinae*	0.23	− 0.12	− 0.24	− 0.29*	− 0.26*	0.16	− 0.05

Type of building: compact [1], free standing [2]; Locality: urban [1], suburban [2]; Place of sampling: walls [0], kitchen floor [1], living-room floor [2], bedroom floor [3], upholstery furniture [4], beds [5], beddings [6]; Month of sampling: 1–12; Weight of sample: in grams; Type of bed: couch [1], sofa [2], bed (mattress) [3]; Type of floor: PVC [1], wooden [2], carpeted [3].

**Table 5:** Relationships observed between housing conditions and the prevalence of mites in dust samples from dwellings of the examined patients in Bytom (south Poland). Results of the Spearman’s rank correlation test analysis (*p < 0.05). Part III.

Number of mites (per 1 g of dust)	Dwelling conditions examined: R Spearman						
	Flowers/plants	Level/floor	Windows	Bed-clothes	Cleaning frequency	Moisture (dampness)	Bedroom temperature
*Dermatophagoides pteronyssinus*	0.24	− 0.20	− 0.20	0.14	0.16	0.33*	− 0.002
*Dermatophagoides farinae*	− 0.09	− 0.27*	0.39*	0.004	0.20	− 0.23	− 0.05
Pyroglyphidae (total)	0.13	− 0.37*	0.14	0.16	0.31*	0.06	− 0.06
Domestic mites (total)	0.15	− 0.38*	0.16	0.16	0.32*	0.05	− 0.08
Total mites	0.15	− 0.38*	0.16	0.16	0.32*	0.05	− 0.08
Live Pyroglyphidae (total)	− 0.13	− 0.13	− 0.28*	0.14	0.13	0.13	0.06
Live domestic mites (total)	0.07	− 0.22	− 0.22	0.16	0.28*	0.07	0.01
Live mites (total)	0.07	− 0.22	− 0.22	0.16	0.28*	0.07	0.01
Live *D. pteronyssinus*	0.19	− 0.20	− 0.22	0.20	0.14	0.34*	0.05
Live *D. farinae*	− 0.49*	0.05	− 0.16	− 0.04	0.01	− 0.27*	0.04

Flowers/plants: absent [1], present [2]; Level: ground floor [0], first floor or higher (I-IX) [1–9]; Type of window frame: wood [1], PVC [2]; Bedclothes: blankets [1], pillows [3]; Cleaning frequency: times per week; Moisture (signs of dampness): absent [1], present [3]; Bedroom temperature: in centigrade degrees.

Moreover, some correlations were observed between parameters of allergic diseases and the prevalence and density of mites in samples from dwellings of the examined allergic patients (Table 6). The severity of asthma was mainly correlated with the density of *D. farinae* per gram of dust, as well as the atopic dermatitis and allergic rhinitis. Higher levels of specific IgE to *D. pteronyssinus* and *D. farinae* were correlated positively with higher densities of *D. farinae*, total pyroglyphids, total domestic mites and total mites per gram of dust, whereas sensitivity on other aeroallergens was positively correlated with the density of *D. pteronyssinus*. Other correlations, both positive and negative, significant or insignificant, are presented in Table 6.

**Table 6:** Correlations observed between parameters of allergic disease and the prevalence of mites in dust samples from dwellings of the examined patients in Bytom (south Poland). Results of the Spearman’s rank correlation test analysis (*p < 0.05).

Prevalence of mites (per 1 g of dust)	Parameters of allergic disease								
	Familial predisposition to atopy	Severity of asthma	Other aeroallergens	Intensification of symptoms	Beginning of disease	Atopic dermatitis	Allergic rhinitis	Level of specific IgE to:	
								DP	DF
*Dermatophagoides pteronyssinus*	− 0.04	0.02	0.27*	0.03	− 0.12	− 0.19	− 0.26*	0.07	0.06
*Dermatophagoides farinae*	0.19	0.26*	0.01	− 0.14	0.10	0.26*	0.27*	0.34*	0.28*
Pyroglyphidae (total)	0.05	0.18	0.16	− 0.11	0.04	0.01	− 0.06	0.33*	0.28*
Domestic mites (total)	0.07	0.19	0.15	− 0.13	0.05	0.02	− 0.06	0.34*	0.29*
Total mites	0.07	0.19	0.15	− 0.13	0.05	0.02	− 0.06	0.34*	0.29*
Live Pyroglyphidae (total)	− 0.01	− 0.37*	0.003	0.01	− 0.21	− 0.30*	− 0.32*	− 0.26*	− 0.27*
Live domestic mites (total)	0.05	− 0.28*	0.08	− 0.12	− 0.27*	− 0.29*	− 0.32*	− 0.14	− 0.14
Live mites (total)	0.05	− 0.28*	0.08	− 0.12	− 0.27*	− 0.29*	− 0.32*	− 0.14	− 0.14
Live *D. pteronyssinus*	− 0.15	− 0.24	0.01	− 0.07	− 0.08	− 0.27*	− 0.40*	− 0.12	− 0.16
Live *D. farinae*	0.16	− 0.26*	− 0.01	0.10	− 0.23	− 0.11	0.21	− 0.24	− 0.20

Familial predisposition to atopy: yes [1], no [0]; Severity of asthma: seasonal [1], moderate [2], severe [3]; Other aeroallergens (scores of skin tests): positive [1], negative [0]; Intensification of symptoms: worse months of a year; Beginning of disease: age of children (in years); Atopic dermatitis: yes [1], no [0]; Allergic rhinitis: yes [1], no [0]; DP: *Dermatophagoides pteronyssinus*; DF: *Dermatophagoides farinae.*

Many significant relationships (positive and negative) were found between housing conditions and characteristics of allergic patients or some parameters of allergic diseases (Wilcoxon signed-rank test). Age and type of the building (old building, brick building) were significantly associated with rhinitis, atopic dermatitis, severity of asthma, higher IgE levels both for *D. pteronyssinus* and *D. farinae*, and reactivity on other allergens (other than domestic mites) (p < 0.001 in all cases). The influence of the presence of pets was also significant for rhinitis (p < 0.001), atopic dermatitis (p < 0.001), severity of asthma (p < 0.05), higher IgE levels both for *D. pteronyssinus* and *D. farinae* (p < 0.001), and reactivity on other allergens (other than domestic mites (p < 0.001).

A higher number of inhabitants, bigger flats and stove heating were also significantly associated with rhinitis, atopic dermatitis, severity of asthma, higher IgE levels both for *D. pteronyssinus* and *D. farinae*, and reactivity on other allergens (other than domestic mites) (p ≤ 0.001 in all cases). Free standing buildings, the presence of flowers, employed house-wives, typical beds (with mattresses), carpeted floors, higher level (floors I-IX), PVC windows, pillows, higher cleaning frequency (times per week) and signs of dampness were significantly associated with rhinitis, atopic dermatitis, higher IgE levels both for *D. pteronyssinus* and *D. farinae*, and reactivity on other allergens (other than domestic mites) (p ≤ 0.01 in all cases) whereas their influence on the severity of asthma was statistically nonsignificant (p > 0.1 in all cases). A higher temperature in the bedrooms was significantly associated with rhinitis, atopic dermatitis, severity of asthma and reactivity on other allergens (other than domestic mites) (p ≤ 0.00005 in all cases) whereas their influence on higher IgE levels for *D. pteronyssinus* and *D. farinae* was statistically nonsignificant (p > 0.1 in both cases).

It should also be stressed that some significant correlations (positive and negative) were found between housing conditions, characteristics of allergic patients and some parameters of allergic diseases (Table 7). The following important significant correlations (p < 0.05) were found (among many others presented in Table 7):

**Table 7:** Correlations observed between housing conditions, characteristics of patients and parameters of allergic disease in the examined patients in Bytom (south Poland). Results of the Spearman’s rank correlation test analysis (*p < 0.05).

	Familial predisposition to atopy	Severity of asthma	Atopic dermatitis	Allergic rhinitis	Other aeroallergens	Intensification of symptoms (month)	IgE level DP	IgE level DF
**Housing conditions**								
Age of building	− 0.42*	− 0.13	− 0.13	− 0.29*	0.36*	0.50*	− 0.26*	− 0.46*
Type of building	− 0.46*	− 0.10	− 0.18	− 0.14	0.31*	0.42*	− 0.17	− 0.41*
Pets	0.03	− 0.11	0.24	− 0.04	− 0.08	− 0.27*	0.08	0.18
Family size	0.33*	0.26*	0.07	− 0.06	− 0.05	0.36*	0.13	0.08
Flat size	− 0.16	− 0.21	0.12	0.15	− 0.32*	0.46*	− 0.12	− 0.14
Type of heating	− 0.14	− 0.07	− 0.27*	− 0.17	0.22	− 0.01	0.10	0.02
Build-up	0.16	− 0.11	0.27*	0.49*	− 0.22	− 0.29*	0.13	0.29*
Flowers/plants	0.35*	0.19	− 0.12	− 0.32*	− 0.25	− 0.19	− 0.05	0.12
House-wife	− 0.65*	− 0.28*	− 0.28*	0.19	− 0.02	0.51*	− 0.36*	− 0.37*
Type of bed	− 0.47*	− 0.60*	− 0.38*	− 0.01	− 0.39*	0.45*	− 0.50*	− 0.47*
Type of floor surface	− 0.07	0.18	− 0.07	− 0.28*	0.06	0.47*	− 0.43*	− 0.40*
Level of dwelling/floor	− 0.18	− 0.10	− 0.37*	− 0.18	0.15	0.38*	− 0.43*	− 0.56*
Window frame	0.53*	0.31*	0.50*	0.37*	− 0.13	− 0.52*	0.31*	0.54*
Bedclothes	− 0.002	0.37*	0.12	− 0.23	0.16	0.20	0.10	− 0.02
Cleaning frequency	0.62*	0.28*	0.19	− 0.14	0.27*	− 0.55*	0.27*	0.23
Moisture/dampness	− 0.35*	− 0.23	− 0.33*	− 0.16	− 0.13	0.21	− 0.44*	− 0.35*
Bedroom temperature	− 0.35*	0.11	− 0.19	− 0.44*	0.37*	0.45*	− 0.26*	− 0.41*
**Characteristics of patients examined**								
Age	0.22	0.25*	0.46*	0.29*	0.27*	− 0.44*	0.36*	0.43*
Sex	0.22	0.11	0.30*	0.32*	0.57*	− 0.02	− 0.05	− 0.08

Age of building: in years; Type of building: block of houses [1], tenement-house [2], brick house (old) [3]; Pets: no pets [0], fish/reptilians [1], birds [2], mammals (cats, dogs, hamsters, rabbits) [3]; Family size: number of inhabitants; Flat size (area occupied by each flat): surface in square meters; Type of heating: central [1], electric [2] or coal-stove [3]; Build-up: compact [1], free standing [2]; Flowers/plants: absent [1], present [2]; House wife: not employed [1], employed [2]; Type of bed: couch [1], sofa [2], bed (mattress) [3]; Type of floor surface (in bedroom): PVC [1], wooden [2], carpeted [3]; Level/floor: ground floor [0], first floor or higher (I-IX) [1–9]; Windows (type of window frame): wood [1], PVC [2]; Bedclothes: blankets [1], pillows [3]; Cleaning frequency: times per week; Moisture/dampness: signs of dampness absent [1], present [3]; Bedroom temperature: in centigrade degrees; Age: age of children (in years); Familial predisposition to atopy: yes [1], no [0]; Severity of asthma: seasonal [1], moderate [2], severe [3]; Atopic dermatitis: yes [1], no [0]; Allergic rhinitis: yes [1], no [0]; Other aeroallergens (scores of skin tests): positive [1], negative [0]; Intensification of symptoms (month): worse months of a year; IgE level DP: levels of specific IgE to *Dermatophagoides pteronyssinus*; IgE level DF: levels of specific IgE to *Dermatophagoides farinae.*

1. familial predisposition to atopy was negatively correlated with the age and type of building, working housewife, type of sleeping accommodation (bed mattresses), higher humidity and higher bedroom temperature, whereas it was positively correlated with higher family size, the presence of flowers/plants, type of windows (PVC) and higher cleaning frequency;
2. severity of asthma was negatively correlated with a working housewife and type of sleeping accommodation (bed mattresses), whereas it was positively correlated with higher family size, type of windows (PVC), bed-clothes (pillows), couches as the type of sleeping accommodations and a higher cleaning frequency;
3. atopic dermatitis was correlated with central heating, free standing houses, not employed house-wife, couches or sofas as types of sleeping accommodation, lower level/floor, type of windows (PVC), lower humidity, and with age (higher) and sex (male).
4. allergic rhinitis was correlated with the lower age of building, free standing buildings, absence of flowers/plants, PVC surface of floors in the bedroom, type of windows (PVC), lower bedroom temperature, and age (higher) and sex (male);
5. sensitivity on other aeroallergens was correlated with older brick buildings, smaller flats, couches and sofas, higher cleaning frequency and a higher bedroom temperature, age (higher) and sex (male).

Other positive and negative correlations, including levels of specific IgE and month of intensification of asthma symptoms, are presented in Table 7.

## Discussion

For the first time in Poland occurrence of house dust mites was investigated in relation to types of dwellings and indoor environmental conditions, possibly affecting the development of asthma in children and adolescents with house dust mite allergy. Pyroglyphid mites usually make up 60–90% of the house dust acarofauna in temperate climate regions throughout the world^12^, also in Poland^4,7,29^. Most often they are found in habitats intimately associated with humans, such as beds, couches, sofas, other upholstery furnitures, clothing, floors and carpets^4,7,9,10,29^. Two mite species *D. pteronyssinus* and *D. farinae* are most often found and most abundant in house dust throughout the world^4,7,9,10,12,14,19,29^. Laboratory culture studies showed that dust mites do not tolerate relative humidity below 60% (or 55% for *D. farinae*)^1–3,5,12,30–33^. However, many house dust surveys suggest a lower limit of relative humidity for dwellings, corresponding to 45% at 20–25 °C. Below these temperatures mites will desiccate and die^1,5,12,30–33^. In certain studies on the other hand, an indoor humidity of 45% RH corresponds to a level of mite density of 100 mites per gram of dust (the threshold value of the risk of exposure to house dust mites) and higher^1–3,12^. It is commonly known that dust mites are more resistant to desiccation at lower temperature values because their Critical Equilibrium Activity (CEA) (or Critical Equilibrium Humidity—CEH) is dependent on the air temperature level, and stays lower with its decrease^1^. Dust mite populations are also found worldwide in dwellings where indoor relative humidity periodically fluctuates on much lower levels than 65% for extended periods of time during each 24 h^1,5,12,26^. At relative humidities ranging between 30–50%, *D*. *farinae* mites are less susceptible to desiccation than *D. pteronyssinus,* which was almost completely absent in the examined samples^5,12^. Ratios of numbers of the particular pyroglyphid dust mite species, especially between *D. pteronyssinus* and *D. farinae*, differ in various regions of the world. Decisive factors influencing their occurrence and abundance are mainly relative humidity and temperature of both outdoor and indoor air^1–4,7,12,34^. It is commonly known that the optimal temperature is higher (25–30 °C) and the optimal humidity is lower (50–75% RH) for *D. farinae* than *D. pteronyssinus*. The first species appears to survive better in dryer habitats than the latter. Lower temperature (15–20 °C) and higher humidity (75–80% RH) favour the survival of *D. pteronyssinus* in mixed laboratory cultures^4,9,10,12,16^. Domination of *D. pteronyssinus* in dwellings appears to be a common tendency at many localities in Poland^7,27^. This tendency may also indicate that dwellings at these localities in our country are more humid. On the other hand, the indoor air ambient humidity, which varies with the degree of ventilation of the dwellings, depends on the buildings’ construction^2,3,17^. Therefore, energy-saving house insulation tends to increase the indoor humidity and may lead to higher house dust mite densities (especially *D. pteronyssinus*)^4,10,26^.

Mites of the families Glycyphagidae and Acaridae are considered much more sensitive to desiccation than pyroglyphids^4,5,12,16,35^. It was also suggested that some domestic mites thrive in very damp conditions. This group includes acarids, glycyphagids and cheyletids. Therefore, the presence and abundance of these mites can be usually used as an indicator of humid environments^4,9,12,16,29^. In general, these mites are not as abundant and frequent in Europe as in the Tropics^6,7,9,10,12,16,29^.

The influence of different housing conditions (both biotic and abiotic) on dust mite populations was widely reviewed by Hart^1^. Some of them such as type of heating, type of mattress, age of furniture, type of bedding, carpets, soft furnishing, soft toys, age of house, number of occupants, presence of pets, floor heating, may influence dust mite populations^1–3,5,7^. Certain recent studies, however, showed weak or no correlation between these environmental factors and mite density^4,9,12,26^.

Environmental factors proved to be significant for the intensification of allergy symptoms in children whose flats were included in the study. A negative correlation between intensification of symptoms and the size of a flat as well as a positive correlation with the number of family members can be associated with an increase in the number of allergens (not only mites) under specific conditions.

An increased exposure to various allergens, including mites, and as a result the intensification of asthma symptoms could have occurred as a result of increased sealing of flats by installing PVC windows, as well as the presence of plants in the flat, sleeping on a couch and using a pillow (significant correlation with asthma severity). Our results are consistent with numerous studies, where the relationship between airborne allergens, air pollution and the development and intensity of bronchial asthma symptoms were found^18,29,36,37^. Furthermore, studies assessing the influence of prevention on the reduction of bronchial asthma symptoms confirm the findings resulting from the obtained data^18,38,39^. At the same time, there are reports showing that prevention based only on the reduction of exposure to mite allergens in the household environment does not provide asthma patients with significant clinical benefits^16,22–24,39–41^.

Rhinitis was significantly associated with the number of *D. farinae*, total house dust mites and total domestic mites per gram of dust. Additionally, the intensity of allergic rhinitis was correlated with environmental conditions. A greater intensity occurred in children living in newer free standing buildings with a lower bedroom temperature, and in buildings with PVC windows, which is certainly related to their greater insulation and the higher level of humidity in rooms; both factors facilitate the reproduction of mites. A lower temperature in bedrooms of children participating in our study was a factor promoting intensification of allergic rhinitis. Two conditions should be met for intensive mite development, one being high temperature (> 20 °C) and the other high humidity (80% of relative humidity). However, it is difficult to compare it with our results as the humidity in the rooms was not measured. Greater intensity of allergic rhinitis was found in children living in free standing buildings. Considering the observations on the behavior of children living in the city, it can be concluded that these children had the opportunity to spend more time outside, in the vicinity of their houses. This kind of behavior promotes the exposure to air pollution, especially fumes released into the atmosphere from car engines. This type of pollution is of similar importance for people living on lower floors. Numerous epidemiological studies have confirmed this mechanism of rhinitis induction and intensification^42,43^. Thus, this relationship does not necessarily have to be induced by the household allergens, including mites.

The intensification of atopic dermatitis was positively correlated with inhabiting free standing buildings and the type of windows installed (e.g. PVC). Window insulation (PVC) increases the exposure to household allergens, including house dust mites. According to literature, airborne allergens, including household ones, can intensify dermal lesions in atopic dermatitis patients^43–50^.

As described above, in accordance with the authors' own materials, the presence of PVC windows was also significant for the severity of asthma and the occurrence of allergic rhinitis. Numerous correlations of the intensity of allergy symptoms with the seasons (particular months) are difficult to explain only by the household environment condition. The relationships between the concentrations of IgE against *D. pteronyssinus* and *D. farinae*, positive correlations with the existence of insulated PVC windows in flats and negative correlations with a place to sleep, deserves special attention. Children sleeping on sofas showed higher IgE concentrations. Moreover, older buildings and stoves are more favourable for the occurrence of both *D. pteronyssinus* and the domestic non-pyroglyphids, whereas new buildings, with central heating systems, for the higher abundance of *D. farinae*. It should also be stressed that *D. farinae* was usually more abundant per gram of dust in samples from couches and sofas whereas *D. pteronyssinus* in bed mattresses^6,7,9^.

## Conclusions

The severity of bronchial asthma in the examined children was associated with those environmental factors influencing the species composition and density of domestic mites in the said children’s dwellings. The relationships also affect other allergic symptoms coexisting with asthma, such as atopic dermatitis and rhinitis. Our attention should be directed to the differences in the influence of particular environmental factors (both abiotic and biotic) on the occurrence and abundance of *D. pteronyssinus* and *D. farinae* in the dwellings of allergic patients. These factors should also be taken into account during medical interviews.

Mite prevalence and density in the examined dwellings suggests associations between density of some mite taxa (per gram of dust) and the following both abiotic or biotic indoor environmental factors: type and age of building (*D. pteronyssinus* with old brick houses, *D. farinae* with block houses and new houses), type of heating (*D. pteronyssinus* and cheyletids with coal-stoves), presence of pets (positive influence on densities of domestic or total mites and live *D. pteronyssinus*), family size and flat sizes (positive influence of smaller families or flats), suburban localisation (on *D. farinae*), place of sampling (beddings and beds on pyroglyphids, domestic and total mites), higher weight of sample (with exception of live *D. farinae*), type of sleeping accommodation (mattresses on live pyroglyphids and couches on *D. farinae*, total pyroglyphids, total domestic mites or total mites), absence of flowers/plants (on live *D. farinae*), lower level/floor of a flat (on *D. farinae, L. destructor*, total pyroglyphids, total domestic mites and total mites), type of windows (PVC on *D. farinae* and wooden on live pyroglyphids), higher cleaning frequency and higher moisture. Generally, older buildings and stoves are more favourable for the occurrence of both *D. pteronyssinus* and the domestic non-pyroglyphids, whereas new buildings, with central heating systems, for the higher abundance of *D. farinae*. It should be also stressed that *D. farinae* was usually more abundant per gram of dust in samples from couches and sofas whereas *D. pteronyssinus* in bed mattresses.

The obtained results regarding domestic acarofauna in the examined dwellings of asthmatic subjects cannot completely account for all variables determining the severity of asthma, as those persons may also be exposed to other indoor allergens. On the other hand, they are also exposed to mite allergens in different outdoor environments; however, these environments are usually home to other mite taxa, not only for the species of the family Pyroglyphidae, known as the house-dust mites. The actual study was performed in 16 flats occupied by 16 patients suffering from bronchial asthma which have agreed to participate in the study. This limited the number of flats examined. As the occurrence and density of mites in dust samples from different places or dwellings may vary to a considerable extent and the number of actually examined flats was limited, the further and more extensive studies are highly desirable. Above all these studies are needed to better clarify the relationships between indoor environmental factors, symptoms of asthma and the species composition of domestic mites from dwellings and other public places in Poland.

## References

[1] Hart BJ (1998). Life cycle and reproduction of house-dust mites: environmental factor influencing mite populations. Allergy.

[2] Korsgaard J (1998). Epidemiology of house-dust mites. Allergy.

[3] Korsgaard J (1998). House-dust mites and asthma. A review on house-dust mites as a domestic risk factor for mite asthma. Allergy.

[4] Solarz K, Seńczuk L, Maniurka H, Cichecka E, Peszke M (2007). Comparisons of the allergenic mite prevalence in dwellings and certain outdoor environments of the Upper Silesia (Southwest Poland). Int. J. Hyg. Environ. Health.

[5] Solarz K (2001). Risk of exposure to house dust pyroglyphid mites in Poland. Ann. Agric. Environ. Med..

[6] Solarz K (2009). Indoor mites and forensic acarology. Exp. Appl. Acarol..

[7] indoor acarofauna of one-family houses (2019). Solarz, K. & Pająk, C. Risk of exposure of a selected rural population in South Poland to allergenic mites. Part I. Exp. Appl. Acarol..

[8] Barnes C (2001). Allergenic materials in the house dust of allergy clinic patients. Ann. Allergy Asthma Immunol..

[9] Boquete M (2006). House dust mite species and allergen levels in Galicia, Spain: a cross-sectional, multicenter, comparative study. J. Invest. Allergol. Clin. Immunol..

[10] Calvo M (2005). Mite allergen exposure, sensitisation and clinical symptoms in Valdivia, Chile. J. Invest. Allergol. Clin. Immunol..

[11] Zeytun E (2015). House dust mites in Erzincan province. Turkiye Parazitol. Derg..

[12] Colloff MJ (2009). Dust Mites.

[13] Asman M, Solarz K, Szilman E, Szilman P (2010). Analysis of expression and amino acid sequence of the allergen Mag 3 in two species of house-dust mites – *Dermatophagoides farinae* and *D. pteronyssinus* (Acari: Astigmata: Pyroglyphidae). Ann. Agric. Environ. Med..

[14] Soltani A, Azizi K, Saleh V, Dabaghmanesh T (2011). The fauna and distribution of house dust mites in residential homes of Bandar Abbas District, Southern Iran. Exp. Appl. Acarol..

[15] Khemili S, Kwasigroch JM, Hamadouche T, Gilis D (2012). Modelling and bioinformatics analysis of the dimeric structure of house dust mite allergens from families 5 and 21: Der f 5 could dimerize as Der p 5. J. Biomol. Struct. Dyn..

[16] Heikal HM (2015). Studies on the occurrence, identification and control of house dust mites at rural houses of Shebin El-Kom locality, Egypt. Pak. J. Biol. Sci..

[17] Thomas WR, Hales BJ, Smith WA (2010). House dust mite allergens in asthma and allergy. Trends Mol. Med..

[18] Kidon MI (2011). Mite component-specific IgE repertoire and phenotypes of allergic disease in childhood: the tropical perspective. Pediatr. Allergy Immunol..

[19] Thongdee D (2011). T cell responses to Der f 2 mite allergens in Thai allergic patients. Health.

[20] Warner JA (2000). Mechanical ventilation and high-efficiency vacuum cleaning: A combined strategy of mite and mite allergen reduction in the control of mite-sensitive asthma. J. Allergy Clin. Immunol..

[21] Arlian LG, Morgan MS, Peterson KT (2008). House dust and storage mite extracts influence skin keratinocyte and fibroblast function. Int. Arch. Allergy Immunol..

[22] Rains N, Siebers R, Crane J, Fitzharris P (1999). House dust mite allergen (Der p 1) accumulation on new synthetic and feather pillows. Clin. Exp. Allergy.

[23] Nam HS, Park CS, Crane J, Siebers R (2004). Endotoxin and house dust mite allergen levels on synthetic and buckwheat pillows. J. Korean Med. Sci..

[24] Custovic A (2000). Synthetic pillows contain higher levels of cat and dog allergen than feather pillows. Pediatr. Allergy Immunol..

[25] Yap JMG, Ching MW, Cabanilla CQ, Ramos JDA, Santos KC (2014). Multiple house dust mite allergen – sensitisation profiles in children with allergic asthma. J. Allergy Ther..

[26] Simpson A, Woodcock A, Custovic A (2001). Housing characteristics and mite allergen levels: to humidity and beyond. Clin. Exp. Allergy.

[27] Obuchowicz A, Solarz K, Nowak W, Szilman E, Pietrzak J, Marek M, Cuber P (2011). Bronchial asthma in children and teenagers and their exposition to house dust mites in urban area in the Upper Silesia. A preliminary study. Ann. Acad. Med. Siles..

[28] Dreborg S, Frew A (1993). Allergen standardization and skin tests. EAACI Position Paper. Allergy.

[29] Kosik-Bogacka DI, Kalisinska E, Henszel L, Kuzna-Grygiel W (2012). Seasonal dynamics of house dust mites in dust samples collected from sleeping places in North-Western Poland. Zoonoses Public Health.

[30] Arlian LG (1992). Water balance and humidity requirements of house dust mites. Exp. Appl. Acarol..

[31] Arlian LG, Confer PD, Rapp CM, Vyszenski-Moher DAL, Chang JSC (1998). Population dynamics of the house dust mites *Dermatophagoides farinae*, *D. pteronyssinus*, and *Euroglyphus maynei* (Acari: Pyroglyphidae) at specific relative humidities. J. Med. Entomol..

[32] Arlian LG, Neal JS, Bacon SW (1998). Survival, fecundity and development of *Dermatophagoides farinae* (Acari: Pyroglyphidae) at fluctuating relative humidity. J. Med. Entomol..

[33] Arlian LG, Neal JS, Vyszenski-Moher DAL (1999). Reducing relative humidity to control the house dust mite *Dermatophagoides farina*e. J. Allergy Clin. Immunol..

[34] Nguyen JL, Schwartz J, Dockery DW (2014). The relationship between indoor and outdoor temperature, apparent temperature, relative humidity, and absolute humidity. Indoor Air.

[35] Fain, A., Guerin, B. & Hart, B. J. Mites and Allergic Disease. (Allerbio, 1990).

[36] Gauderman WJ (2004). The effect of air pollution on lung development from 10 to 18 years of age. N. Engl. J. Med..

[37] Panettieri RA (2020). Onset of effect, changes in airflow obstruction and lung volume, and health related quality of life improvements with benralizumab for patients with severe eosinophilic asthma: phase IIIb randomized, controlled trial (SOLANA). J. Asthma Allergy.

[38] Huss K (2001). House dust mite and cockroach exposure are strong risk factors for positive allergy skin test responses in the Childhood Asthma Management Program. J. Allergy Clin. Immunol..

[39] Hachim MY (2020). Confounding patient factors affecting the proper interpretation of the periostin level as a biomarker in asthma development. J. Asthma Allergy.

[40] Sears MR (2003). A longitudinal, population-based, cohort study of childhood asthma followed to adulthood. N. Engl. J. Med..

[41] Morgan W (2004). Inner-City Asthma Study Group: Results of a home-based environmental intervention among urban children with asthma. N. Engl. J. Med..

[42] Corver K (2006). House dust mite allergen reduction and allergy at 4 years: follow up of the PIAMA-study. Pediatr. Allergy Immunol..

[43] Bousquet J (2008). Allergic rhinitis and its impact on asthma (ARIA) 2008. Allergy.

[44] Ricci G (2000). Effect of house dust mite avoidance measures in children with atopic dermatitis. Br. J. Dermatol..

[45] Van Cauwenberge P (2000). Consensus statement on the treatment of allergic rhinitis. Allergy.

[46] Capristo C, Romei I, Boner AL (2004). Environmental prevention in atopic eczema dermatitis syndrome (AEDS) and asthma: avoidance of indoor allergens. Allergy.

[47] Gelbard C, Hebert AA (2008). New and emerging trends in the treatment of atopic dermatitis. Patient Prefer. Adherence.

[48] Shek LP (2010). Specific profiles of house dust mite sensitisation in children with asthma and in children with eczema. Pediatr. Allergy Immunol..

[49] Shekariah T, Kalavala M, Alfaham M (2011). Atopic dermatitis in children: A practical approach. Paediatr. Child Health.

[50] Park KH (2018). Sensitisation to various minor house dust mite allergens is greater in patients with atopic dermatitis than in those with respiratory allergy disease. Clin. Exp. Allergy.

